# An Unusual Presentation of a Posterior Mediastinal Schwannoma Associated with Traumatic Hemothorax

**DOI:** 10.1155/2015/175645

**Published:** 2015-05-07

**Authors:** Ruchi Amin, Brett H. Waibel

**Affiliations:** Department of Surgery, Division of Trauma and Surgical Critical Care, The Brody School of Medicine, East Carolina University, Greenville, NC 27834, USA

## Abstract

Schwannomas of the thoracic cavity are typically an asymptomatic, benign neurogenic neoplasm of the posterior mediastinum. In this case, we present a traumatic hemothorax as the initial presentation for a previously undiscovered mediastinal mass. The patient presented with shortness of breath and right-sided chest pain after being struck in the chest with a soccer ball. An operative exploration was pursued due to persistent hemothorax with hemodynamic instability despite resuscitation and adequate thoracostomy tube placement. The intraoperative etiology of bleeding was discovered to be traumatic fracture of a large hypervascular posterior mediastinal schwannoma. Surgical resection is the treatment of choice for these tumors. Specific serological markers do not exist for this tumor, and radiographic findings can be variable, so tissue diagnosis is of importance in differentiating benign from malignant schwannomas, as well as other posterior mediastinal tumors. However, most patients have excellent survival following complete resection.

## 1. Introduction

The schwannoma is a benign neurogenic neoplasm, with peak incidence in the third through fifth decades of life, affecting men and women equally. While most are asymptomatic, the most common complaint is invasion of intercostal nerve, bone, and chest wall, or cough and dyspnea from compression of the tracheobronchial tree [1]. We present a case in which initial presentation was uncontrolled hemorrhage after direct traumatic injury to the chest wall.

## 2. Case Presentation

**Figure 1:**
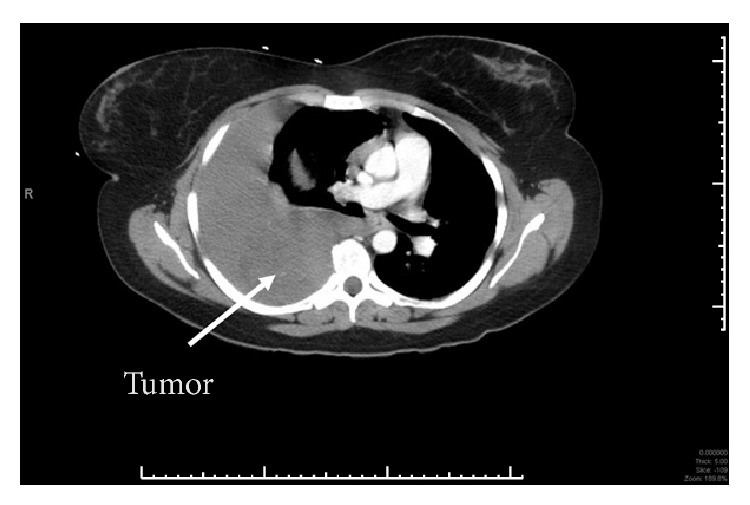
Outside hospital CT scan showing hemothorax and unrecognized schwannoma.

**Figure 2:**
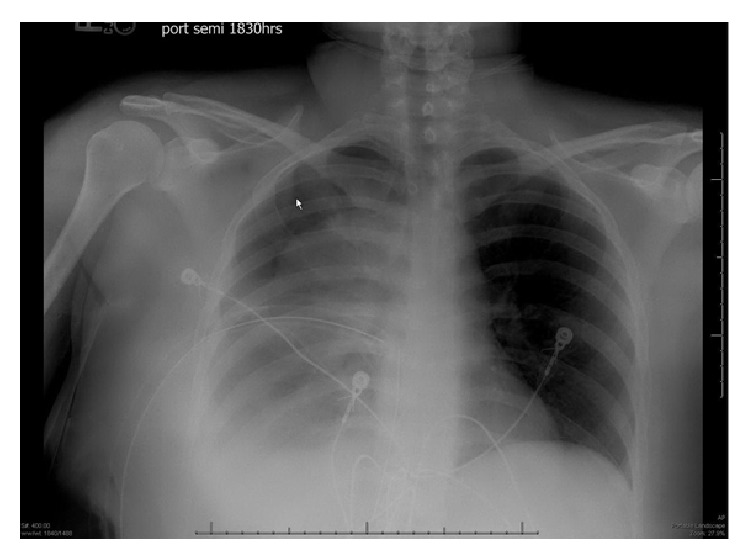
Chest X-ray obtained at trauma center showing persistent hemothorax despite chest tube placement at outside hospital.

A 34-year-old woman was transferred to our Level I Trauma Center with shortness of breath and right-sided chest pain after being hit in the chest with a soccer ball. Truncal CT (Figure 1) from the outside hospital showed a right hemothorax, for which she had a chest tube placed. Prior to arrival, the patient had received four units of pRBC and three liters of crystalloid. Upon arrival, the patient was hypotensive, was tachycardic, and had drained two liters of blood over the two hours since placement of the chest tube. A chest X-ray (Figure 2) showed a persistent large hemothorax. The patient was taken emergently to the operating room for a right thoracotomy given her hemodynamics. A large (10 × 10 cm) nonpulsatile soft tissue mass was found in the mid-right chest cavity along the posterior edge of the pleural space with active hemorrhage from an injury. Attempts at hemostasis using electrocautery and topical hemostatic agents were unsuccessful. The mass was excised with help from thoracic surgery, and a frozen section confirmed histology consistent with a schwannoma. Hemostasis was achieved, and there was no evidence of injury to the azygos vein or adjacent lung. The lung reexpanded without issue, a thoracostomy tube was placed, and the incision was closed. She was extubated on postoperative day (POD) 1. The chest tube was removed on POD 3, with discharge home the next day. The patient had an uneventful recovery with significant improvement in pain and resumption of daily activities upon her follow-up visit. Final pathology showed a benign schwannoma.

## 3. Discussion

The posterior compartment of the mediastinum is bordered anteriorly by the pericardium and trachea, posteriorly by the anterior border of the spine, and contains the descending aorta, esophagus, thoracic duct, and vagus nerve. Posterior neurogenic tumors originate from the neural crest and are further classified as peripheral, sympathetic, or paraganglionic in origin. Though significantly less common, tumors of lymphatic, vascular, and mesenchymal origin may also occur. The most common pediatric mediastinal neurogenic tumors are the ganglioneuroma and neuroblastoma. While the ganglioneuroma is a benign tumor, the neuroblastoma is highly malignant. In the adult population, the neurofibroma and schwannoma are most common, with the schwannoma being the most common posterior mediastinal neurogenic neoplasm.

Symptoms associated with posterior compartment neurogenic tumors result from compression of the airway, esophagus, right heart, and great veins. Malignant tumors have the added potential of invasion into the tracheobronchial tree, lungs, esophagus, superior vena cava, pleura, and chest wall, presenting as cough, stridor, dyspnea, hemoptysis, dysphagia, pleural effusion, and/or superior vena cava syndrome. With adjacent nerve involvement, there is risk for development of hoarseness, neuropathic pain, diaphragmatic paralysis, or Horner's syndrome. Paraneoplastic syndromes, such as those seen with autonomic nerve cell tumors, are rarely seen with these tumors. Rarely, even the benign tumors can also present with massive hemothorax. In a review article by Miura et al., of seven patients with spontaneous hemothorax, five tumors were benign (one benign schwannoma, four neurofibromas) and the remaining two were malignant (one malignant schwannoma, one neurofibrosarcoma) [2].

The initial workup of a posterior mediastinal mass involves radiographic evaluation, with the majority of asymptomatic masses discovered incidentally on posteroanterior (PA) and lateral radiographs. Additional radiologic findings include enlargement of the neural foramina (creating a dumbbell shaped lesion), scalloping of posterior vertebral bodies, erosion of the ribs, pleural effusion, and scoliosis. Disruption of the azygoesophageal recess is a nonspecific finding seen in both middle and posterior mediastinal masses. Posterior masses generally have sharp margins due to their interface with the lung [3]. CT imaging of the mass is useful in determining the exact location and its relationship to adjacent structures and may aid in differentiating tissue densities between cystic, vascular, and solid masses. Due to high contrast resolution and multiplanar capability, MRI is the preferred modality of imaging as it provides better evaluation regarding the nature and extent of intraspinal involvement towards the neural and vertebral foramen. The limitations of MRI compared to CT are the limited radiographic evaluation of calcifications and poorer spatial resolution [4]; therefore, while an MRI is preferable it is not always necessary. Recent studies have offered ultrasound guided FNA as a viable diagnostic modality for accessible mediastinal lesions [5], allowing for formal diagnosis without the potential complications from more invasive procedures such as CT-guided biopsy, mediastinoscopy, or video-assisted thoracoscopic surgery which offer no benefit in the management of these tumors. There has been some concern for using EUS as a method for FNA as the amount of tissue for cytologic interpretation may not be adequate; however, with the addition of immunostains, some have argued that a conclusive diagnosis can be made [5]. PET scans have shown the ability to distinguish between malignant peripheral nerve sheath tumors and neurofibromas with high accuracy but are not as helpful in distinguishing between benign and malignant peripheral nerve sheath tumors [6].

Surgical resection is the primary treatment of choice in most neurogenic tumors, including the schwannoma. As these are generally benign tumors, efforts should be directed towards a minimally invasive resection, even when they arise as synchronous lesions or an unusual location [7]. Rarely, schwannomas originate from the vagus, phrenic, or any part of the intercostal nerve and should be removed through a nerve-sparing technique [8]. Radiation therapy may be used postoperatively to control residual disease in malignant schwannomas, but its benefit is unknown. No known chemotherapeutic regimens are effective against these tumors. Since serologic markers are absent and characteristic imaging abnormalities are variable, tissue pathology and immunohistochemistry are required for a diagnosis. Schwannomas are composed of spindle cells with twisted nuclei, amphophilic cytoplasm, and rare mitoses [9]. Patients with neurofibromatosis are likely to display a variant form called plexiform schwannoma. Another variant, the melanocytic schwannoma, has a pronounced brownish cytoplasmic pigment and malignant potential. The malignant schwannoma, the most dangerous variant, is a soft gray-pinkish tumor with central necrosis and microscopically consists of sheets of pleomorphic spindle cells with numerous mitotic figures and necrotic areas. Malignant schwannomas may also exhibit a variety of cellular components such as clusters of epithelial cells; mucin-secreting glands; and even mesenchymal features such as bone, cartilage, or skeletal muscle. Patients who undergo resection of a benign schwannoma are generally followed up to monitor wound healing and resumption of daily activities. Routine follow-up of patients who undergo curative resection of a malignant neoplasm has not demonstrated a survival benefit in randomized controlled trials due to the variety and infrequency of malignant mediastinal tumors [10]. Overall, patients with benign schwannoma have excellent survival following complete resection, whereas those with malignant tumors have a poorer prognosis [11].
